# Diffusion Tensor Imaging for Assessment of Response to Neoadjuvant Chemotherapy in Patients With Breast Cancer

**DOI:** 10.18383/j.tom.2016.00271

**Published:** 2016-12

**Authors:** Lisa J. Wilmes, Wen Li, Hee Jung Shin, David C. Newitt, Evelyn Proctor, Roy Harnish, Nola M. Hylton

**Affiliations:** 1 Department of Radiology and Biomedical Imaging, University of California, San Francisco, California; and; 2 Department of Radiology and Research Institute of Radiology, Medical Imaging Laboratory, Asan Medical Center, University of Ulsan College of Medicine, Seoul, South Korea

**Keywords:** diffusion tensor imaging, apparent diffusion coefficient, fractional anisotropy, neoadjuvant therapy, breast cancer

## Abstract

In this study, the prognostic significance of tumor metrics derived from diffusion tensor imaging (DTI) was evaluated in patients with locally advanced breast cancer undergoing neoadjuvant therapy. DTI and contrast-enhanced magnetic resonance imaging were acquired at 1.5 T in 34 patients before treatment and after 3 cycles of taxane-based therapy (early treatment). Tumor fractional anisotropy (FA), principal eigenvalues (λ1, λ2, and λ3), and apparent diffusion coefficient (ADC) were estimated for tumor regions of interest drawn on DTI data. The association between DTI metrics and final tumor volume change was evaluated with Spearman rank correlation. DTI metrics were investigated as predictors of pathological complete response (pCR) by calculating the area under the receiver operating characteristic curve (AUC). Early changes in tumor FA and ADC significantly correlated with final tumor volume change post therapy (ρ = −0.38, *P* = .03 and ρ = −0.71, *P* < .001, respectively). Pretreatment tumor ADC was significantly lower in the pCR than in the non-pCR group (*P* = .04). At early treatment, patients with pCR had significantly higher percent changes of tumor λ1, λ2, λ3, and ADC than those without pCR. The AUCs for early percent changes in tumor FA and ADC were 0.60 and 0.83, respectively. The early percent changes in tumor eigenvalues and ADC were the strongest DTI-derived predictors of pCR. Although early percent change in tumor FA had a weak association with pCR, the significant correlation with final tumor volume change suggests that this metric changes with therapy and may merit further evaluation.

## Introduction

There is increasing clinical interest in the use of magnetic resonance imaging (MRI) techniques to evaluate tumors in patients with locally advanced breast cancer (LABC) who are receiving (preoperative) neoadjuvant chemotherapy (NACT). The ability to predict response at an early time point in treatment could potentially allow for modification of treatment to improve efficacy and decrease morbidity.

Although change in tumor size has been shown to be a surrogate predictor of response to chemotherapy (1), changes in tumor morphology tend to occur later in therapy and after biological effects such as changes in tumor microvasculature or cellular density (2, 3). Diffusion-weighted imaging (DWI) is an MRI technique that is being investigated as a potential biomarker of early response to treatment. DWI has the advantage of not requiring administration of a contrast agent, and echo planar imaging-DWI enables relatively short image acquisition times. DWI uses diffusion-sensitizing gradients to characterize the magnitude of water motion within tissue and thus indirectly provide information about tissue cellularity and microstructure. The apparent diffusion coefficient (ADC) is a quantitative measure of water mobility calculated from DWI images.

Previous DWI studies have found that the ADC in breast tumors is reduced relative to the ADC in normal tissue, and this difference is correlated with an increase in cell density in tumors (4, 5). DWI has been shown to increase diagnostic accuracy of malignant breast lesions in combination with dynamic contrast-enhanced (DCE)-MRI (6, 7). In a DWI study of patients with breast cancer undergoing NACT, an increase in tumor ADC was shown to precede a decrease in tumor size (3). Other DWI studies of patients receiving NACT have found that pretreatment tumor ADC and early change in tumor ADC were associated with final tumor volume response (8) or pathological complete response (pCR) (9).

More recently, the breast has been studied with diffusion tensor imaging (DTI), which is sensitive to both the magnitude and direction of water motion. DTI uses a minimum of 6 noncollinear gradient directions to characterize the full diffusion tensor and calculate the 3 main eigenvalues of the diffusion tensor (λ1, λ2, and λ3), allowing for the estimation of the directionality (anisotropy) of water diffusion within tissue in addition to mean diffusivity (ADC). Fractional anisotropy (FA) is a commonly used DTI-derived metric that reflects the degree of directionality of diffusion. In tissues where diffusion is isotropic, that is, λ1 = λ2 = λ3, the theoretical value of FA is zero, whereas if diffusion occurs principally along 1 direction and is highly restricted in the other directions, FA will approach the maximum value of 1.

An initial study of breast cancer using DTI found that FA was lower in breast tumors than in normal breast fibroglandular tissue, and that FA provided increased diagnostic accuracy over ADC alone (10). It was hypothesized that the lower FA in tumors may be attributable to a disruption of the organized structure of normal breast parenchyma, characterized by a network of ducts and lobules supported by stroma, caused by the disorganized growth and increased cellularity of tumors (10). Subsequent DTI studies of breast have reproduced the finding of lower FA in tumors (11, 12); however, other studies have found no difference in FA between tumor and normal tissue (13, 14). To date, little has been reported on the effects of treatment on DTI-measured metrics in breast tumors.

In this preliminary work, the prognostic significance of DTI was evaluated in patients with LABC undergoing NACT. In particular, the associations between tumor DTI metrics and the following two measures of tumor response were investigated:

(1) change in tumor volume at the end of NACT; and
(2) tumor pCR, which is a clinical measure of tumor response.

## Methods

### Patient Population

Patients with biopsy-confirmed LABC undergoing NACT consisting of 12 weekly cycles of taxane-based therapy followed by Adriamycin–Cytoxan (AC) treatment, were enrolled in institutional review board-approved, Health Insurance Portability and Accountability Act-compliant clinical trials at our institution. All patients gave informed consent. MRI was performed for each patient on the following 4 separate visits: before the start of NACT (MRI_1_, pretreatment); after the third weekly cycle of paclitaxel treatment (MRI_2_, early treatment); after the final cycle of paclitaxel treatment but before the start of AC treatment (MRI_3_, inter-regimen); and after the completion of AC treatment and before surgery (MRI_4_, presurgery). Patients were scanned using a standard DCE-MRI protocol, and a subset of patients was also scanned with DTI. In total, 40 women who were scanned with both DCE-MRI and DTI were included in this study. The data from 6 patients were excluded because of DTI scan protocol deviations; so, the final study cohort included 34 patients.

### MRI Data Acquisition

All MRI data were acquired on a 1.5 T GE Signa HDx scanner (GE Healthcare, Waukesha, Wisconsin) using an 8-channel bilateral phased array breast coil (Invivo Corp., Gainesville, Florida [formerly Sentinelle Medical, Toronto, Canada]). Bilateral DCE-MRI images were acquired in the axial orientation using a 3-dimensional fast gradient echo sequence with the following parameters: repitition time (TR) = 7 (ms), echo time (TE) = 4.2 ms, flip angle = 10°, and Array Spatial Sensitivity Encoding Technique (ASSET) parallel imaging acceleration factor = 2. Field of view varied from 280 to 360 mm to achieve full bilateral coverage. Patients received 0.1 mmol/kg body weight of gadopentetate dimeglumine (Magnevist, Bayer Healthcare Pharmaceuticals, Berlin, Germany) contrast agent.

Bilateral, axial diffusion tensor images were acquired after DCE-MRI, using a fat-suppressed single-shot echo planar DWI sequence with the following parameters: TR = 6000 ms, TE = 69.6 ms, field of view = 400 × 400 mm, acquisition matrix = 128 × 128, slice thickness = 3 mm, slice skip = 0 mm, number of signal averages = 6, and ASSET factor = 2. Diffusion gradients were applied in 6 directions with b values = 0 and 600 s/mm^2^. The image acquisition time was 4.3 minutes.

### DTI Data Analysis

Parametric maps of the 5 rotationally invariant DTI metrics—mean diffusivity (D_avg_), also referred to as ADC; maximum tensor eigenvalue (λ1); intermediate tensor eigenvalues (λ2); minimum (λ3) tensor eigenvalues; and FA—were calculated from DTI data based on the methods of Basser and Pierpaoli (15), using in-house software developed in IDL (ITT Visual Information Solutions, Boulder, Colorado). The ADC reflects the magnitude of water mobility and was calculated using equation (1) as follows:

(1) $$ADC=\frac{\lambda_{1}+\lambda_{2}+\lambda_{3}}{3}{mm}^{2}/\sec ond$$

FA is a unitless measure that reflects the degree of directionality of diffusivity, and was calculated using equation (2) as follows:

(2) $$FA=\frac{\sqrt{{\left(\lambda_{1}-\lambda_{2}\right)}^{2}+{\left(\lambda_{2}-\lambda_{3}\right)}^{2}+{\left(\lambda_{3}-\lambda_{1}\right)}^{2}}}{\sqrt{2}\sqrt{\lambda_{1}^{2}+\lambda_{2}^{2}+\lambda_{3}^{2}}}$$

An ROI for each tumor was manually defined on a representative slice of the MRI_1_ and MRI_2_ diffusion tensor images by a radiologist with 7 years of experience in evaluating breast MRIs. The ROIs were subsequently reviewed by an MRI scientist with 13 years of experience in evaluating breast MRIs and one with 1 year of experience, to ensure that ROIs encompassed only tumor tissue; this resulted in modification of 3 of the ROIs. Tumor ROIs were drawn to encompass areas that were hyperintense on b = 600 s/mm^2^ combined images and hypointense on corresponding ADC maps. Enhancing areas on corresponding DCE-MRI subtraction images (precontrast subtracted from postcontrast) were also used to guide ROI selection. Clip artifacts and areas of necrosis were excluded from the ROIs. Mean and median tumor FA; λ1, λ2, and λ3; and ADC values were calculated from the corresponding parametric maps for each ROI. The mean difference between the maximum and minimum eigenvalues (λ1–λ3) for tumor was also calculated. Tumor DTI metrics were calculated for time points MRI_1_ and MRI_2_, and the early treatment percent changes in DTI metrics [(MRI_2_ − MRI_1_)/MRI_1_) × 100] were also calculated. Additionally, an ROI was drawn for each patient in normal-appearing breast fibroglandular tissue on the contralateral breast, whenever possible on the same DTI slice in a similar location within the unaffected breast (similar anterior/posterior offset and right/left offset) as the tumor. These ROIs were used to calculate mean FA and ADC values for normal breast tissue.

### Response Assessment: Tumor Final Volume Presurgery and Tumor Pathology Postsurgery

Functional tumor volume (FTV) for each imaging visit was calculated from DCE-MRI using a previously described semiautomated segmentation method for calculating the volume of all tumor voxels that exceeded a percent enhancement threshold of 70% at the second postcontrast time point (16). The FTV analysis was performed by 2 MRI scientists with 10+ years of experience, and all segmentations were reviewed and approved by the designated breast radiologist at our institution. The early treatment percent changes in FTV (ΔFTV_2_, from MRI_1_ to MRI_2_) and the final percent change in FTV, after completion of NACT and before surgery (ΔFTV_NACT_, from MRI_1_ to MRI_4_), were calculated for statistical analysis.

The pathological response to NACT was assessed after surgery by gross and microscopic examination of the excised tumor specimens, and then classified into 2 groups—pCR or non-pCR. In our study, pCR was defined as no residual invasive disease (17). Information on tumor histological grade, estrogen and progesterone receptor, HER-2/neu, and lymph node metastasis was obtained from histopathological reports.

### Statistical Analysis

An unpaired *t*-test was used to estimate the difference between mean ages in pCR and non-pCR groups, based on the assumption that age followed a normal distribution. The Fisher exact test was used to estimate associations between histological subtype, hormone receptor, HER-2/neu, lymph node metastasis, and pCR versus non-pCR.

The Shapiro–Wilk test was used to test the normality of tumor DTI-measured metrics and MRI volume. Differences in DTI-measured metrics were evaluated between tumor and normal tissue with the Wilcoxon signed rank test. Spearman's rank-order correlation coefficient ρ was used to assess the association between MRI-derived tumor metrics (FA, eigenvalues, ADC, and volume) measured at MRI_1_ and MRI_2_, (including early percent changes between MRI_1_ and MRI_2_) and the final percent tumor volume change at MRI_4_ (ΔFTV_NACT_). The Mann–Whitney U-test was used to evaluate differences in MRI-measured tumor metrics between the pCR and non-pCR groups. Data were expressed as median with interquartile ranges.

A receiver operating characteristic (ROC) curve was used to assess the performance of MRI-measured tumor metrics in differentiating pCR from non-pCR, and the areas under the ROC curve (AUCs) were calculated. Further, the 95% confidence intervals were calculated for AUCs with 2000 stratified bootstrap replicates. Two-tailed statistical tests were always used, and findings with a *P*-value <.05 were considered statistically significant. Statistical analysis was performed using R: a language and environment for statistical computing (Vienna, Austria) (18).

## Results

### Patients

A total of 34 lesions in 34 patients (mean age, 48.1 years; range, 32–70 years) who underwent definitive surgery after completing NACT were evaluated with DTI in addition to DCE-MRI from September 2010 to June 2012. Patient characteristics are shown in Table 1. Histopathological assessment of surgical specimens collected after completion of NACT found that 25 of 34 (73.5%) patients had residual invasive disease and were classified as non-pCR, whereas 9 (26.5%) were classified as pCR with no evidence of invasive disease. Of the 9 patients with pCR, 6 (66.7%) showed no evidence of malignant cells and 3 (33.3%) showed ductal carcinoma in situ only. Pathology showed invasive ductal carcinoma in 33 patients (97.1%) and mixed ductal and lobular carcinoma in 1 patient (2.9%). No significant difference in patient age was found between the pCR and non-pCR groups. Significant associations with pCR outcome were found for estrogen receptor status and lymph node metastasis. Due to the small sample size, we did not adjust for estrogen receptor status or lymph node metastasis in our statistical analysis.

**Table 1.** Clinical Characteristics of Patients and Lesions (N = 34)

Characteristics	pCR (n = 9)	Non-pCR (n = 25)	*P*-Value
Age (years), mean (range)	51.9 (39–70)	46.8 (32–64)	0.14
Menopausal status			0.65
Premenopausal	5	13	
Perimenopausal	0	4	
Postmenopausal	4	8	
Surgery method			0.70
Mastectomy	3	11	
Breast-conserving surgery	6	14	
Histological subtype			1
Invasive ductal carcinoma	9	24	
Mixed ductal and lobular carcinoma	0	1	
Estrogen receptor			0.026
Negative	0	7	
Positive	5	16	
NA	4	2	
Progesterone receptor			0.06
Negative	2	12	
Positive	3	11	
NA	4	2	
HER-2/neu			0.31
Negative	2	12	
Positive	2	7	
Indeterminate (FISH)	1	2	
NA	4	4	
Lymph node metastasis			0.006
Negative	3	12	
Positive	2	13	
NA	4	0	

Abbreviations: pCR, pathological complete response; NA, not available.

### FA and ADC in Tumor and Normal Breast Fibroglandular Tissue: Full Cohort

The mean pretreatment (MRI_1_) FAs for tumor and normal breast fibroglandular tissue for the full cohort were 0.18 ± 0.06 and 0.21 ± 0.06, respectively. The mean early treatment (MRI_2_) FAs for tumor and normal fibroglandular tissue were 0.21 ± 0.05 × 10^−3^ mm^2^/s and 0.22 ± 0.07, respectively. The FA of tumor was significantly lower than that of fibroglandular tissue before treatment (MRI_1_; *P* = .03), but not at early treatment (*P* = .4). The FA of tumor of the full cohort was significantly higher at MRI_2_ than at MRI_1_ (*P* = .003).

The mean pretreatment (MRI_1_) ADCs for tumor and normal fibroglandular tissue for the full cohort were 1.15 ± 0.15 × 10^−3^ mm^2^/s and 2.05 ± 0.25 × 10^-3^ mm^2^/s, respectively. The mean early treatment (MRI_2_) ADCs for tumor and normal fibroglandular tissue for the full cohort were 1.51 ± 0.22 × 10^−3^ mm^2^/s and 2.07 ± 0.23 × 10^−3^ mm^2^/s, respectively. The ADC of tumor was significantly lower than that of fibroglandular tissue at both time points (*P* < .0001), and the ADC of tumor at MRI_2_ was significantly higher than that at MRI_1_ (*P* < .0001).

### Correlation of Tumor MRI Metrics and Final Tumor Volume Change: Full Cohort

Spearman rank correlation test found no statistically significant associations between pretreatment (MRI_1_) tumor MRI metrics and percent FTV change after NACT (ΔFTV_NACT_); results are shown in Table 2. At the early treatment time point (MRI_2_), significant correlations with ΔFTV_NACT_ were found for λ1 (*P* = .005), λ2 (*P* = .006), λ1–λ3 (*P* = .01), and ADC (*P* < .001), with ρ ranging from −0.42 to −0.47.

**Table 2.** Association of Pre- and Early Treatment DTI- and DCE-Derived Metrics With %Change in Functional Tumor Volume Post Treatment (ΔFTV_NACT_) Assessed With Spearman Rank Correlation (ρ)

MRI MetricTumor	MRI_1_		MRI_2_		%ChangeMRI_1_ to MRI_2_	
	ρ	(*P*-Value)	ρ	(*P*-Value)	ρ	(*P*-Value)
FA	−0.004	(.98)	−0.21	(.24)	−0.38	(.03)^a^
λ1	0.09	(.6)	−0.47	(.005)^a^	−0.65	(<.001)^a^
λ2	0.28	(.1)	−0.46	(.006)^a^	−0.73	(<.001)^a^
λ3	0.21	(.2)	−0.33	(.06)	−0.68	(<.001)^a^
λ1–λ3	0.05	(.8)	−0.42	(.01)^a^	−0.46	(.007)^a^
ADC	0.21	(.22)	−0.45	(<.001)^a^	−0.71	(<.001)^a^
FTV	0.14	(.42)	0.38	(.03)^a^	0.39	(.02)^a^

Abbreviations: DCE, dynamic contrast-enhanced; DTI, diffusion tensor imaging; FTV, functional tumor volume; FA, fractional anisotropy; ADC, apparent diffusion coefficient; MRI, magnetic resonance imaging.

^a^Statistically significant correlations (*P* < .05).

Statistically significant correlations with ΔFTV_NACT_ were also found for the early percent changes in all tumor MRI metrics, with strong and significant correlations found for the following diffusivity measures: λ1, λ2, λ3, and ADC, with ρ ranging from −0.65 to −0.73 and *P* <.001 for all measures (Table 2). Moderate and significant correlations were found for early percent changes in λ1–λ3 (ρ = −0.43, *P* = .007) and FA (ρ = −0.38, *P* = .03) and for early percent change in tumor volume, ΔFTV_2_ (ρ = 0.39, *P* = .02).

No significant association was found between tumor FA and tumor ADC at either MRI_1_ (ρ = 0.03, *P* = .86) or MRI_2_ (ρ = 0.2, *P* = .3). However, a statistically significant correlation was found between the early percent change in tumor ADC and the early percent change in FA (ρ = 0.42, *P* = .01).

### Comparison of Pre- and Early Treatment Tumor MRI Metrics Between pCR and Non-pCR Groups

At the pretreatment time point (MRI_1_), patients in the pCR group had significantly lower tumor λ2 (*P* = .05) and ADC (*P* = .04) than those in the non-pCR group (Table 3). Although λ1 and λ3 were also lower in the pCR group, these differences were not significance. At the early treatment time point (MRI_2_), no significant differences in tumor MRI metrics were found (Table 3).

**Table 3.** Comparison of Tumor FA, Principal Eigenvalues, ADC, and Volume Between pCR and Non-pCR Groups for MRI_1_, MR_2_ and %Change MRI_1_ to MRI_2_

MRI Tumor Metric	pCR		Non-pCR		*P*-Value
	M	IQR	M	IQR	
MRI_1_ (Pretreatment)					
FA	0.15	0.14, 0.19	0.17	0.15, 0.20	.62
λ1	1.25	1.11, 1.35	1.35	1.26, 1.45	.06
λ2	1.04	0.96, 1.09	1.14	1.08, 1.20	.05^a^
λ3	0.87	0.77, 0.95	0.96	0.89, 1.00	.09
λ1–λ3	0.30	0.26, 0.45	0.40	0.33, 0.46	.3
ADC	1.07	0.98, 1.10	1.15	1.09, 1.19	.04^a^
FTV	8.3	4.8, 19.3	24.4	8.0, 36.1	.20
MRI_2_ (Early treatment)					
FA	0.21	0.18, 0.23	0.21	0.17, 0.21	.82
λ1	1.77	1.66, 2.05	1.74	1.58, 1.98	.5
λ2	1.56	1.38, 1.63	1.45	1.33, 1.64	.8
λ3	1.15	1.13, 1.31	1.18	1.07, 1.31	.6
λ1–λ3	0.62	0.51, 0.86	0.60	0.47, 0.77	.7
ADC	1.50	1.40, 1.68	1.43	1.33, 1.65	.59
FTV	7.4	2.5, 16.2	6.1	3.4, 17.2	.89
%Change MRI_1_ to MRI_2_					
FA	28.0	12.3, 32.1	16.5	5.0, 29.0	.49
λ1	55.9	47.2, 62.0	30.1	21.9, 42.0	.004^a^
λ2	52.6	43.8, 57.6	25.6	21.1, 42.5	.008^a^
λ3	44.2	35.0, 45.7	21.3	14.5, 30.8	.009^a^
λ1–λ3	89.2	69.7, 116.6	45.9	35.2, 75.0	.07
ADC	53.2	43.9, 57.2	26.4	20.0, 40.5	.002^a^
FTV	−46.6	−72.8, −10.7	−54.8	−72.5, −26.2	.42

Abbreviations: pCR, pathological complete response; IQR, interquartile range; MRI, magnetic resonance imaging; FA, fractional anisotropy; ADC, apparent diffusion coefficient; FTV, functional tumor volume.

Data represent the median value of the metric (M) and the interquartile range (IQR).

^a^Statistically significant differences with *P* < .05.

**Figure 1.**
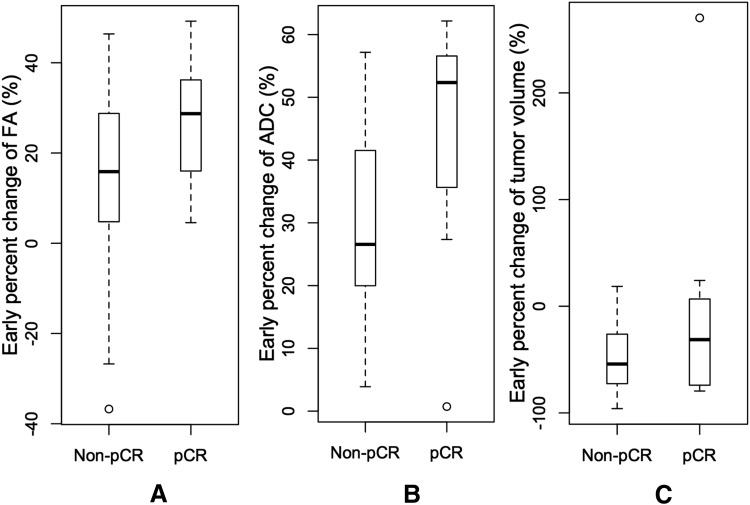
Box plots of early percent change in tumor fractional anisotropy (FA), apparent diffusion coefficient (ADC), and volume between pathological complete response (pCR) and non-pCR groups. Early percent change of FA was not significantly different between the 2 groups (*P* = .49) (A). Early percent change in ADC in the pCR group was significantly higher than that in the non-pCR group (*P* = .002) (B). No significant difference was found for early percent change in tumor volume between the pCR and non-pCR groups (C).

The early percent changes in the following tumor MRI metrics were found to be statistically significantly higher in the pCR versus the non-pCR group: λ1 (*P* = .004), λ2 (*P* = .008), λ3 (*P* = .009), and ADC (*P* = .002) (Table 3). No statistically significant differences between the pCR group and the non-pCR group were found for the early percent change in tumor FA (*P* = .42) or FTV (*P* = .49). The early percent changes in tumor FA, ADC, and FTV for the pCR and non-pCR groups are summarized in the plots in Figure 1. Although the early percent change in the pCR group appears higher than that for the non-pCR group for all 3 MRI metrics, only the percent ADC change was significantly different between the 2 groups.

**Figure 2.**
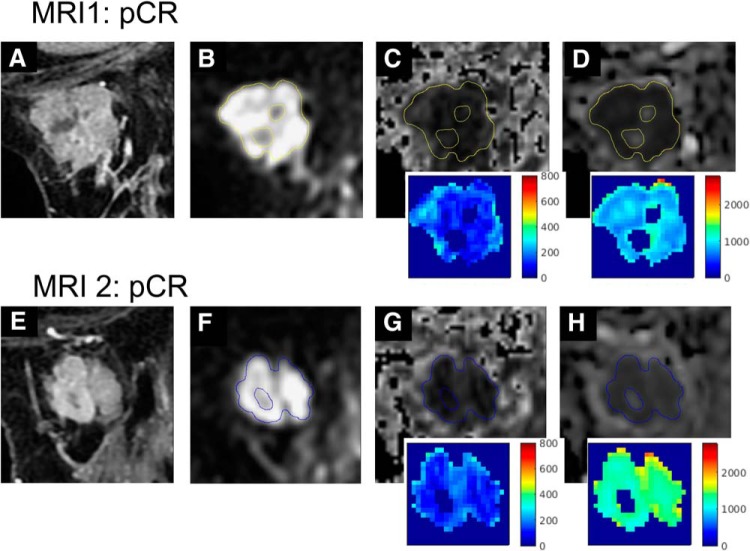
Example images from a patient with locally advanced breast cancer (LABC) who was categorized as pCR based on postsurgical pathology. Dynamic contrast-enhanced magnetic resonance imaging (DCE-MRI) subtraction images (precontrast subtracted from early postcontrast) and diffusion tensor imaging (DTI) images (b = 600, FA map, and ADC map) are shown for the pretreatment time points (A, B, C, and D) and early treatment time points (E, F, G, and H). The tumor region of interest (ROI) is indicated by the contour line on the DTI b = 600 images and FA and ADC maps, and colorized maps of the FA and ADC values within the tumor ROI are also shown.

**Figure 3.**
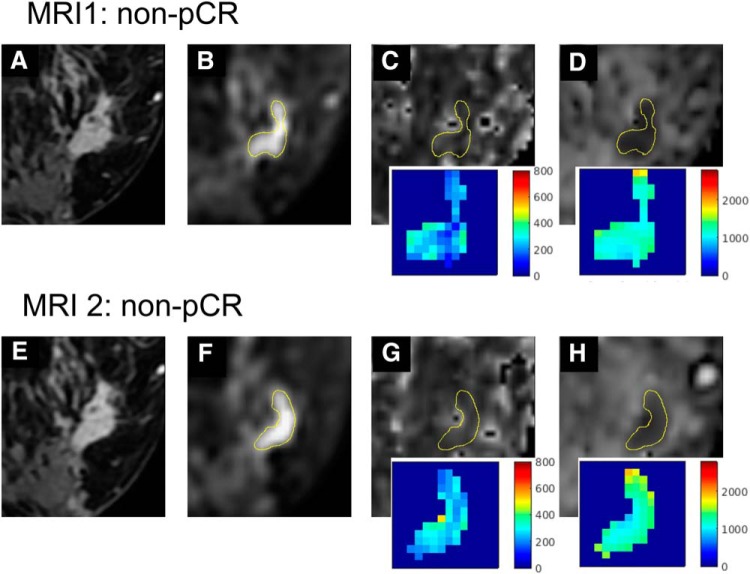
Example images from a patient with LABC who was categorized as non-pCR based on postsurgical pathology. DCE-MRI subtraction images (precontrast subtracted from early postcontrast) and DTI images (b = 600, FA map, and ADC map) are shown for the pretreatment time points (A, B, C, and D) and early treatment time points (E, F, G, and H). The tumor ROI is indicated by the contour line on the DTI b = 600 images and FA and ADC maps, and colorized maps of the FA and ADC values within the tumor ROI are also shown.

Representative images from DCE-MRI (early postcontrast) and DTI are shown for MRI_1_ and MRI_2_ time points for a tumor that was classified as pCR (Figure 2) and for a tumor that was classified as non-pCR (Figure 3). For DTI, the b = 600 image and the corresponding FA and ADC parametric maps are shown with the tumor ROI delineated. For these 2 examples, a visible change in FA between MRI_1_ and MRI_2_ is difficult to appreciate for either the pCR tumor (Figure 2, C and G) or the non-pCR tumor (Figure 3, C and G). For the tumor ADC maps, the values throughout the tumor generally increase between MRI_1_ and MRI_2_ for the pCR tumor (Figure 2, D and H); however, no such change is visibly apparent in the non-pCR tumor (Figure 3, D and H).

### Association of Pre- and Early Treatment Tumor MRI Metrics With pCR

The AUCs and confidence intervals from ROC curve analysis of tumor MRI metrics for the pre- and early treatment time points are shown in Table 4. The following were the pretreatment MRI metrics that had statistically significant associations with pCR: λ2 and ADC, with AUCs of 0.72 and 0.74. Pretreatment λ1 and λ3 AUCs were in a similar range, but these did not reach statistical significance. None of the MRI metrics showed a significant association with pCR for the early treatment (MRI_2_) time point. The early percent changes (MRI_1_ to MRI_2_) in λ1, λ2, λ3, and ADC were all statistically significantly associated with pCR, with AUCs of 0.82 0.80, 0.79, and 0.83 respectively. FA measures were not statistically significantly associated with pCR, with the highest AUC of 0.58 found for early percent change in FA. Similarly, early time point measures for FTV did not achieve significance, with the highest AUC value for tumor volume being 0.65 for pretreatment FTV.

**Table 4.** AUCs of Pre- and Early Treatment MRI-Derived Tumor Metrics for Predicting pCR

MRI Tumor Metric	MRI_1_		MRI_2_		%Change MRI_1_ to MRI_2_	
	AUC	CI	AUC	CI	AUC	CI
ADC	0.74^a^	0.51, 0.96	0.56	0.34, 0.79	0.83^a^	0.61, 1.00
FA	0.56	0.31, 0.81	0.53	0.30, 0.76	0.58	0.34, 0.82
FTV	0.65	0.42, 0.88	0.52	0.30, 0.74	0.60	0.35, 0.85
λ1	0.72	0.50, 0.93	0.57	0.34, 0.80	0.82^a^	0.61, 1.00
λ2	0.72	0.49, 0.95	0.54	0.32, 0.76	0.80^a^	0.57, 1.00
λ3	0.70	0.46, 0.93	0.44	0.24, 0.64	0.79^a^	0.56, 1.00
λ1–λ3	0.72	0.50, 0.93	0.57	0.34, 0.80	0.71	0.47, 0.95

Abbreviations: AUC, areas under the ROC curve; CI, confidence interval; MRI, magnetic resonance imaging.

^a^AUCs >0.5, with *P* < .05.

## Discussion

In this preliminary study, DTI-derived tumor metrics (FA, principal eigenvalues and ADC) and DCE-MRI tumor volume metrics were measured in patients with LABC undergoing NACT before and after 3 cycles of taxane-based therapy. The associations between these tumor MRI metrics and treatment response as characterized by final FTV change post NACT (ΔFTV_NACT_) and pCR were evaluated. Early percent change in all tumor MRI metrics showed a significant correlation with ΔFTV_NACT_. Of the tumor metrics evaluated, the early percent changes in tumor principal eigenvalues and ADC had the strongest associations with pCR.

The pretreatment tumor FA, principal eigenvalues, and ADC values measured in this patient cohort were within the ranges measured in other diagnostic DTI studies of the breast using the same MRI field strength and b values (10, 12). Consistent with previous breast DTI studies (10–12), the pretreatment ADCs in this patient cohort were found to be significantly lower in tumors than in normal tissue. The lower tumor ADC values are also consistent with previous DWI studies of breast cancer (4, 19). Tissues with higher cell density are expected to have more restricted water mobility, and the lower ADC in breast tumors has been shown to correlate with increased cell density (20). The pretreatment tumor FA in this cohort was significantly lower than that in normal breast fibroglandular tissue. This is consistent with the hypothesis that breast tumors represent a disruption of normal breast tissue structure and is in agreement with some DTI studies of breast tumors (10–12, 21). Although other DTI studies in breast have reported no differences in FA between tumor and normal fibroglandular tissue (13, 14), these were diagnostic studies in which tumor sizes are generally not as large as in the setting of LABC. Measurement of DTI metrics in larger tumors may be less affected by partial volume effects from adjacent tissue that can hinder differentiation of tumor from normal tissue.

The association between pre- and early treatment tumor MRI metrics and change in FTV after NACT was evaluated because FTV change has been shown to be a predictor of recurrence-free survival (1, 22). At the early treatment time point (MRI_2_), the tumor diffusivity measures, λ1, λ2, λ1–λ3, and ADC, had statistically significant correlations with ΔFTV_NACT_. FTV measured at MRI_2_ also had a significant, but weaker, correlation with final tumor volume change.

Interestingly, significant correlations were found between the early percent changes in all tumor MRI metrics and ΔFTV_NACT_. The strongest significant (negative) correlations were found for early percent change in tumor λ1, λ2, λ3, and ADC. The correlation between early percent change in ADC and post-treatment tumor volume is in agreement with some previous DWI studies of patients with breast cancer undergoing NACT (23), although other DWI studies of NACT have not found such a correlation (3, 24). Moderate statistically significant correlations were found for λ1–λ3, FA, and ΔFTV_2_.

The association between MRI tumor metrics and pCR was evaluated because pCR has been established as an independent prognostic marker for overall survival in breast cancer and is currently the gold standard for assessing response to NACT (25). Patients displaying complete disappearance of the tumor at surgical pathology after NACT have favorable overall survival rates and decreased risk of recurrence (26–28). At the pretreatment time point (MRI_1_) λ2 and ADC were statistically significantly lower in the pCR group. The tumor ADC finding is consistent with other published work that found that lower pretreatment breast tumor ADC measured by DWI was associated with treatment response in patients undergoing NACT (8, 23); however, not all studies have found this association (3, 24).

The early percent changes in tumor λ1, λ2, λ3, and ADC were all significantly higher in the pCR group than in the non-pCR group. The early percent change in tumor ADC for the pCR group (53.2%) in this study was comparable with results from another study, which found a significantly greater increase in tumor ADC in patients with breast cancer in the pCR versus non-pCR groups after 4 cycles of 5-fluorouracil epirubicin cyclophosphamide NACT (9). The early percent increases in tumor λ1 and λ2 in the pCR group were also comparable with the early percent increase in tumor ADC found in the pCR group. The early-treatment percent increases found for tumor λ1, λ2, and λ3 had fairly narrow ranges for both the pCR and non-pCR groups, (56% to 44% and 30% to 21%, respectively), suggesting that the 3 orthogonal eigenvectors increased symmetrically, at least during the early treatment phase.

Although the early percent change in tumor FA was greater for the pCR group than for the non-pCR group, this difference was not significant. Both tumor ADC and FA were statistically significantly higher in the full cohort post treatment, and early changes in both metrics were significantly associated with ΔFTV_NACT_; however, only the change in ADC was significantly associated with pCR. Although it can be hypothesized that neoadjuvant treatment may affect tissue microstructure and organization in a way that may be reflected in changes in both tumor ADC and FA, to date, little has been published on the effects of NACT on breast tumor DTI metrics such as FA and eigenvalues.

One recent abstract using DTI to assess early changes in ADC and FA in patients with advanced breast cancer receiving AC NACT found a significant increase in tumor ADC, but no significant change in tumor FA, after 1 cycle of therapy (29). Another DTI abstract measured changes in tumor ADC and FA at early and later time points in patients with breast cancer receiving NACT, and found that although ADC was significantly increased in responders at the early treatment time point, a significant increase in FA was not observed until the later treatment time point (30). These findings suggest that changes in tumor FA may occur at different time points compared with tumor ADC and may be dependent on the type of treatment.

The lack of significant association of FA parameters with pCR status may also be reflective of the higher within-subject coefficient of variation that has been found for FA (11.4%) compared with that for ADC (4.5%) (31). In addition, the criteria used to draw the tumor ROI may affect FA values more than ADC values. In the study by Balzter et al. (11), evaluating DTI in breast lesions and normal breast fibroglandular tissue, two types of tumor ROIs were drawn: one encompassing the whole tumor on the b = 1000 images and the other including only the brightest areas from the original whole-tumor ROI. The FA measured from the tumor ROI type that encompassed the whole tumor was not significantly different from normal breast tissue FA, whereas the FA measured from the tumor ROI type that encompassed only the brightest areas was significantly lower than the FA from the normal breast tissue. When both types of tumor ROIs were applied to ADC maps, the mean tumor ADCs were slightly different; however, significant differences between tumor ADC and normal breast tissue ADC were found for both tumor ROI types. Thus, the criteria used to select the tumor ROI may also affect the sensitivity of the FA measurement.

There are several limitations to the current study. One limitation is the relatively small number of patients scanned with DTI. In addition, a limitation of standard single shot-echo planar imaging-DWI sequences is that the spatial resolution is relatively low (ie, larger voxels) compared with gradient echo sequences commonly used for DCE-MRI. As a consequence, tissue partial volume effects may limit the ability of DTI metrics, in particular those measuring anisotropy, to detect response. It has been suggested that imaging techniques with higher spatial resolution may improve the predictive value of DTI in breast (11). Another potential limitation is the use of a single slice ROI to characterize each tumor rather than a multi-slice ROI that may better reflect the full range of tumor values for DTI metrics. Finally, the timing of the early treatment measurement may not have been optimal for evaluating changes in tumor FA.

In conclusion, this study found that of all the DTI metrics evaluated, the early percent changes in tumor principal eigenvalues and ADC had the strongest associations with pCR, and these measures were stronger predictors than early changes in FTV. Although early percent change in tumor FA had a weak association with pCR, the significant correlation of early percent change in FA with final tumor volume change suggests that this parameter may provide additional information on tumor response and may merit further evaluation in a larger patient cohort. Future DTI studies may benefit from assessing changes at a later time point during treatment, as changes in FA and ADC may not occur at the same rate. The utilization of more advanced DTI techniques, with reduced distortion and/or improved spatial resolution, that are now becoming commercially available, may also improve the ability to detect differences in FA between tissues and treatment-induced changes in FA within a tissue over time.
